# Comparison of standard polymer formula versus short peptide formula in sepsis patients with acute gastrointestinal injury

**DOI:** 10.3389/fnut.2025.1682020

**Published:** 2025-10-20

**Authors:** Youquan Wang, Yanhua Li, Yuhan Zhang, Dong Zhang

**Affiliations:** Department of Critical Care Medicine, The First Hospital of Jilin University, Changchun, China

**Keywords:** sepsis, enteral nutrition, acute gastrointestinal injury, standard-polymeric formulas, short-peptide formulas

## Abstract

**Background:**

To investigate the selection of enteral nutrition (EN) formulas for critically ill sepsis patients with acute gastrointestinal injury (AGI) grade I-II.

**Methods:**

We conducted a retrospective cohort study on critically ill sepsis patients with AGI grades I-II. The primary outcomes were EN caloric adequacy on day 7, calories and protein gain from EN within 3–7 days, and the incidence of gastric retention and diarrhea after EN administration. We performed a subgroup analysis based on whether patients initiated EN within 48 h.

**Results:**

In the early EN subgroup, caloric adequacy, calories and protein gain of EN on day 7 of the short-peptide group was higher than that of the standard-polymeric group (59.1% vs. 27.3%, *p* = 0.001; 624[0, 936] vs. 0[−360, 480], *p* = 0.001; 24[0, 38] vs. 0[−14.4, 19.2], *p* = 0.003, respectively), and the incidence of gastric retention (18.2% vs. 36.4%, *p* = 0.03) and diarrhea (9.1% vs. 25.5%, *p* = 0.02) were lower in the short-peptide group than in the standard-polymeric group. However, in the delayed EN subgroup, the caloric adequacy of EN on day 7 of the short-peptide group was lower than that of the standard-polymeric group (28.6% vs. 43.5%, *p* = 0.02), calories and protein gain from EN were lower in the short-peptide group than in the standard-polymeric group (960[480, 1,200] vs. 1,080[720, 1,440], *p* = 0.04; 38.4[19.2, 50.4] vs. 43.2[28.8, 57.6], *p* = 0.04, respectively).

**Conclusion:**

In sepsis patients with AGI grades I–II, short-peptide formulas may be considered for early EN initiation (≤48 h), while standard-polymer formulas may be an option for late EN initiation (>48 h). Exploratory results need to be interpreted with caution and await verification of these findings through high-quality research.

## 1 Introduction

Sepsis and septic shock are common reasons for admission to the intensive care unit (ICU) (1). These conditions can potentially lead to intestinal ischemia, impaired intestinal barrier, and even dysbiosis, resulting in acute gastrointestinal injury (AGI) in critically ill patients. The Working Group on Abdominal Problems of the European Society of Intensive Care Medicine (ESICM) defines AGI as gastrointestinal dysfunction due to acute illness in intensive care patients (2). Studies have shown that critically ill patients without AGI experience lower mortality than those with AGI (3–8). Therefore, it is imperative to pay attention to the AGI in patients with sepsis.

Early enteral nutrition (EN) can produce beneficial physiological effects in critically ill patients via the downregulation of systemic immune responses, reduction of oxidative stress, and maintenance of gut microecology, leading to improved patient outcomes (9–14). Some recent studies have shown that EN is associated with lower AGI grades in patients with AGI and lower mortality in patients with sepsis (15–17). Hence, EN plays a vital role in sepsis patients with AGI. In line with the 2021 international guidelines for the management of sepsis and septic shock, early initiation of EN within 72 h is recommended for adult patients with sepsis or septic shock who are suitable for enteral feeding (18). Unfortunately, the guideline did not provide specific recommendations regarding EN formulas for patients with sepsis.

Guidelines recommend the routine use of standard polymeric formulas in critically ill patients (12). However, it remains unclear whether short-peptide formulas can leverage their pre-digested properties to reduce the burden of digestion and absorption and thereby benefit sepsis patients with AGI (19–21). Therefore, this exploratory study aimed to investigate the effects of these two EN formulas on clinical outcomes in critically ill sepsis patients with AGI grades I and II.

## 2 Materials and methods

### 2.1 Study design and participants

**Figure 1:**
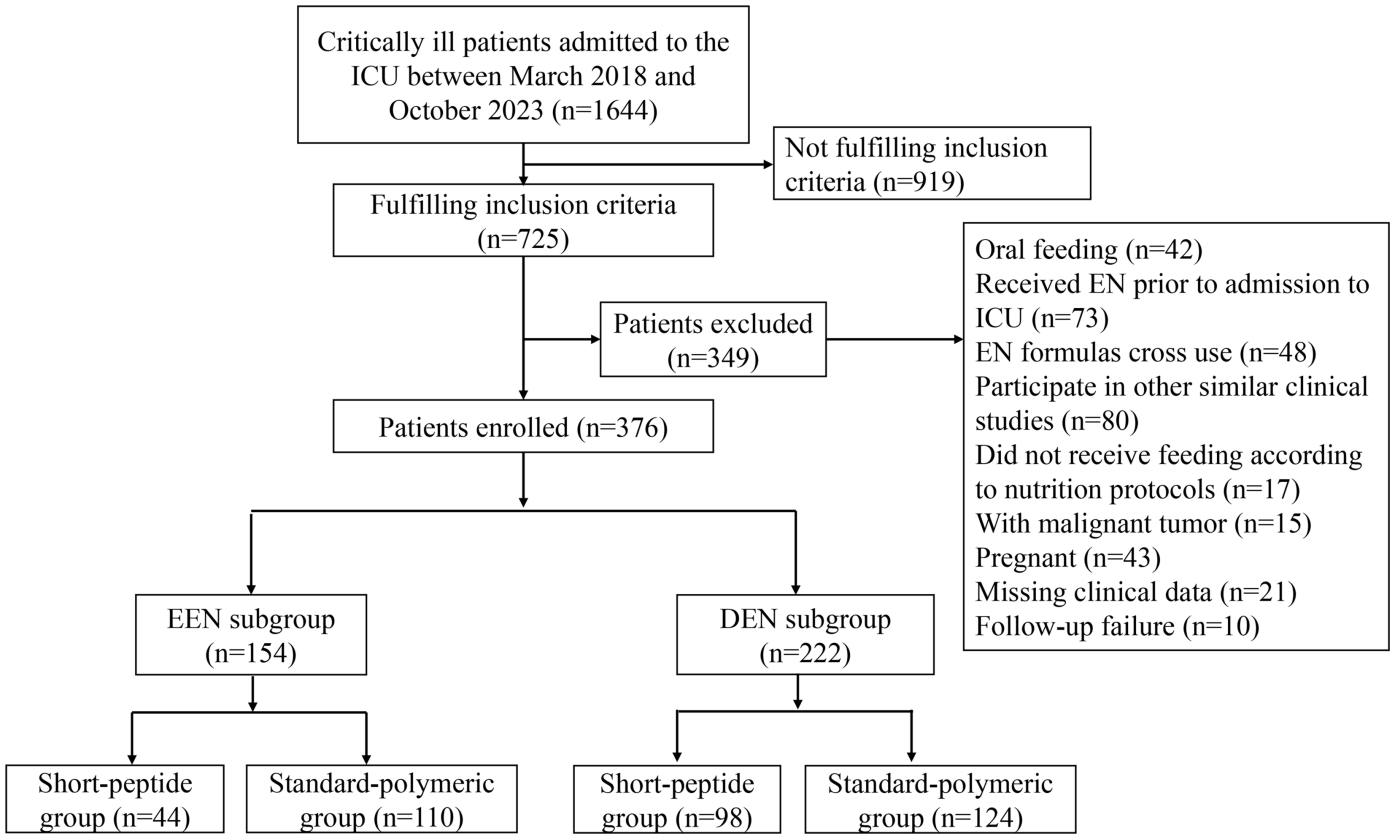
Patient inclusion flowchart.

We conducted a retrospective cohort study on adult patients with AGI and sepsis admitted to the ICU of our hospital (The comprehensive ICU of the third class and has a total of 56 beds in 3 treatment areas) between March 2018 and October 2023 (Figure 1). The inclusion criteria of this study included: (1) age ≥ 18 years; (2) met the diagnostic criteria of Sepsis-3 (22); (3) admitted to the ICU for ≥7 days; (4) AGI grades I-II (AGI grade I: risk of developing gastrointestinal dysfunction or failure; AGI grade II: gastrointestinal dysfunction) proposed by the 2012 ESICM guideline (2); and (5) short-peptide formulas or standard-polymeric formulas used within 7 days of ICU admission. The exclusion criteria of this study were as follows: (1) received oral feeding, (2) EN was administered prior to ICU admission, (3) did not receive feeding according to nutrition protocols, (4) EN formulas cross use, (5) had participated in other similar clinical studies, (6) had malignant tumors, (7) were pregnant, (8) missed clinical data, (9) follow-up failure. According to the type of EN formulas used by critically ill patients, they were assigned to a short-peptide formulas group and a standard-polymeric formulas group.

The 2018 European Society for Clinical Nutrition and Metabolism (ESPEN) guidelines divided the clinical stage of patients in the ICU into the Ebb and flow phases; the first 7 days of the flow phase are called the acute phase (9), and the acute phase is divided into the early period (metabolic instability and severe increase in catabolism, within 48 h after injury) and late period (a significant muscle wasting and a stabilization of the metabolic disturbances, 48 h after injury). Considering the differences in the severity of severe disease, the severity of infection, and the presence or absence of shock, different EN initiation times were selected. To reduce bias due to baseline differences, we set up a subgroup analysis based on this guideline. Patients were assigned to the early EN (EEN) subgroup (those who received EN within 48 h) and delayed EN (DEN) subgroup (those who received EN within day 3–7). Different degrees of AGI were shown in Table 1, and the EN startup ratios of the EEN and DEN subgroups were shown in Supplementary Figure S1.

**Table 1:** Different degrees of AGI.

AGI grades	Diagnostic criteria
AGI grade I	The function of the GI tract is partially impaired, expressed as GI symptoms related to a known cause and perceived as transient.
AGI grade II	The GI tract cannot perform digestion and absorption adequately to satisfy the nutrient and fluid requirements of the body. There are no changes in the patient’s general condition related to GI problems.
AGI grade III	Loss of GI function, where restoration of GI function is not achieved despite interventions and the general condition is not improving.
AGI grade IV	AGI has progressed to become directly and immediately life-threatening, worsening MODS and shock.

AGI, acute gastrointestinal injury; GI, gastrointestinal; MODS, multiple organ dysfunction syndrome.

In accordance with the relevant guidelines and regulations, all methods employed in this retrospective cohort study were conducted in compliance with ethical standards and approved by the Ethics Committee of the First Hospital of Jilin University (No. 2022-483). Informed consent was waived by the Ethics Committee of the First Hospital of Jilin University for this retrospective study, as it did not involve human participants or tissue samples.

### 2.2 Nutrition protocols

Clinicians in our department had implemented customized nutrition protocols for each severely ill AGI patient according to ESICM guidelines (2). For example, patients with AGI grades I–II and severe disease were initiated on an EF dose of 20 mL/h (2), with caloric and protein targets set at 25 kcal/kg/day and 1.2–2.0 g/kg/day, respectively (23), as recommended by guidelines, actual body weight was used for patients with body mass index (BMI) < 25 kg/m^2^, whereas adjusted body weight (AdjBW) was used for BMI ≥ 25 kg/m^2^. AdjBW was calculated as ideal body weight (IBW) + 0.25 × (Actual − IBW) and that IBW was calculated as 0.9 × height (cm) − 100 for males and 0.9 × height (cm) − 106 for females (9). Appropriate supplemental parenteral nutrition was provided if enteral routes fail to meet nutritional goals within a short timeframe. Initiating EN at a full dose at the start of feeding was prespecified as a protocol violation. Because this was a retrospective study, temporary EN dose reductions or interruptions during the subsequent course of feeding (e.g., in response to gastrointestinal symptoms or persistent shock) were considered routine clinical care and were not classified as protocol deviations.

Additionally, GI symptoms are closely monitored to adjust EF dosing. Patients who had lost or did not follow their nutritional protocols were excluded from this study. The clinician selects the short-peptide formulas (1 kcaL/mL, 20 g/500 mL of protein) or standard-polymeric formulas (1.5 kcaL/mL, 30 g/500 mL of protein) according to their preference and patient’s condition. The comparison of the characteristics of the two formulas is detailed in Supplementary Table S1. All patients were fed with continuous infusion.

### 2.3 Clinical data collection

Collect patient clinical data from electronic medical records, such as primary diagnosis (most important cause or disease leading to admission to the ICU), EN initiation time, acute physiology and chronic health evaluation (APACHE) II scores, sequential organ failure assessment (SOFA) scores, nutrition risk screening (NRS) scores, modified NUTRIC (mNUTRIC) scores, AGI grades, and serum albumin levels. All scores related to disease severity and nutritional status and AGI grades were calculated from the clinical data of severely ill patients within the first 24 h of ICU admission.

The patients’ daily intakes of protein and calories from EN, ICU length of stay, hospital length of stay, hospitalization cost, ventilator-free days, ICU and 28-day mortality, were recorded, and adverse gastrointestinal events during their ICU stay, if it occurred, were also obtained.

### 2.4 Primary and secondary outcomes

The primary outcomes were caloric adequacy of EN (≥80% of target calories) on day 7. The secondary outcomes were calories and protein gain from EN within 3–7 days (calories or protein fed through EN on the 7th day minus calories or protein fed through EN on the 3rd day), the incidence of gastric retention (a single volume ≥ 200 mL), and diarrhea (loose stools ≥ 3 times per day or liquid stools with a stool weight > 250 mL/day or > 200–250 g/day) (2) after EN administration, daily caloric EN/(EN + Parenteral nutrition (PN)) ratios, ICU and 28-day mortality, ICU and hospital length of stay, ventilator-free days and hospitalization cost.

### 2.5 Sample size calculation

To the best of our knowledge, no previous studies have directly compared short-peptide and standard polymeric formulas in sepsis patients with AGI. Based on a prior study in AGI patients (20), we assumed probabilities of achieving 80% of the caloric target by day 7 to be 80% (short-peptide group) and 50% (standard polymeric group), with a 1:1 allocation ratio. Using these proportions, a sample size of 72 patients (36 per group) was calculated (*α* = 0.05, power = 0.80) via PASS software. Because our prespecified analyses compared the two formulas separately within the EEN and DEN subgroups, the overall planned sample size was 144 patients (EEN: 72 [36 + 36]; DEN: 72 [36 + 36]). Given the exploratory nature of this study, this sample size is adequate for preliminary insights.

### 2.6 Statistical analysis

The statistical software used was SPSS 26.0 (Armonk, NY, United States: IBM Corp). Normality of continuous variables was assessed using the Shapiro–Wilk test (two-sided *α* = 0.05) within each study group. Continuous variables are defined as mean ± standard deviation if they were normally distributed; otherwise, median values and interquartile ranges M (P25, P75) were represented. Differences between the two groups were determined using *t*-test for normally distributed continuous variables and the Mann–Whitney *U* test for nonparametric data. Categorical data were compared between the two groups using the chi-square test (or Fisher’s exact test when expected counts were <5). The Spearman correlation coefficient and variance inflation factor were performed to detect collinearity among the variables.

For prespecified secondary outcomes, we controlled the false discovery rate (FDR) at 5% using the Benjamini–Hochberg procedure; for transparency, we report uncorrected *p*-values together with FDR-adjusted *p*-values in the Supplementary Table S7. Parameters with *p* < 0.1 in the univariate analysis were entered into the multiple logistic regression analysis to identify the predictors for caloric adequacy of EN on day 7. The confounders considered in the multivariate regression analysis were based on the results of the univariate logistic regression for different subgroups (EEN and DEN). The confounders included in each subgroup’s multivariate logistic regression were different, as outlined in Supplementary Tables S2, S3. Kaplan–Meier survival analysis was performed to estimate the 28-day cumulative survival. Statistical significance for the primary outcome was set at two-sided *p* < 0.05 (no multiplicity adjustment).

Due to the retrospective nature of this study, significant differences in baseline characteristics between the early enteral nutrition (EEN) and delayed enteral nutrition (DEN) groups precluded their combined analysis. Therefore, we separately compared the effects of short-peptide and standard polymeric formulas within each subgroup (EEN and DEN) to explore their clinical outcomes.

## 3 Results

### 3.1 Baseline characteristics and initial severity of illness

Of the 1,644 patients screened for eligibility, a total of 376 patients who met the inclusion criteria and did not meet the exclusion criteria were eventually included in the study. The number of excluded patients and the reasons are as follows: 919 patients did not fulfill the inclusion criteria, 42 patients received oral feeding, 73 patients received EN prior to ICU admission, 48 patients were cross-used EN formulations, 80 patients had participated in other similar clinical studies, 17 patients did not feeding follow the nutritional protocols (start with a full-dose feeding of 40-50 mL/h), 15 patients had malignant tumors, 43 patients were pregnant, 21 patients had missing clinical data, and 10 patients were not followed-up. Therefore, 376 patients were evaluated for the effect of the short-peptide and standard-polymeric formulas on the feeding outcome and clinical outcome of severely ill patients with sepsis and AGI grades I–II (Figure 1). Baseline patient characteristics of the EEN and DEN subgroups are presented in Table 2.

**Table 2:** Demographic data and clinical characteristics of patients at baseline.

Characteristics	EEN subgroup				DEN subgroup			
	All (*n* = 154)	Short-peptide (*n* = 44)	Standard-polymeric (*n* = 110)	*P*-value	All (*n* = 222)	Short-peptide (*n* = 98)	Standard-polymeric (*n* = 124)	*P*-value
Sex, male, *n* (%)	98 (63.6)	32 (72.7)	66 (60.0)	0.14	136 (61.3)	56 (57.1)	80 (64.5)	0.26
Age, mean ± SD, years.	62.3 ± 15.5	65.7 ± 16.1	60.9 ± 15.1	0.08	58.6 ± 17.9	59.5 ± 18.4	57.9 ± 17.5	0.52
Actual body weight, median (IQR), kg	65 (60–70)	70 (60–75)	65 (60–70)	0.09	65 (60–70)	65 (60–70)	65 (60–70)	0.26
BMI, mean ± SD	22.8 ± 4.0	22.9 ± 3.1	22.8 ± 4.3	0.82	22.8 ± 3.6	22.2 ± 3.6	22.8 ± 3.1	0.15
Underlying conditions, *n* (%)								
Diabetes mellitus	59 (38.3)	17 (38.6)	42 (38.2)	0.96	88 (39.6)	32 (32.7)	56 (45.2)	0.06
Hypertension	68 (44.2)	22 (50.0)	46 (41.8)	0.36	128 (57.7)	60 (61.2)	68 (54.8)	0.34
Chronic obstructive pulmonary disease	32 (20.8)	11 (25.0)	21 (19.1)	0.41	52 (23.4)	22 (22.4)	30 (24.2)	0.76
Coronary heart disease	45 (29.2)	13 (29.5)	32 (29.1)	0.96	56 (25.2)	24 (24.5)	32 (25.8)	0.82
Primary diagnosis, *n* (%)								
Neurologic	38 (24.7)	11 (25.0)	27 (24.5)	0.95	18 (8.1)	6 (6.1)	12 (9.7)	0.34
Respiratory	46 (29.9)	15 (34.1)	31 (28.2)	0.47	22 (9.9)	8 (8.2)	14 (11.3)	0.44
Cardiovascular	29 (18.8)	8 (18.2)	21 (19.1)	0.90	38 (17.1)	14 (14.3)	24 (19.4)	0.32
Gastrointestinal	18 (11.7)	6 (13.6)	12 (10.9)	0.63	86 (38.7)	44 (44.9)	42 (33.9)	0.09
Multi trauma	12 (7.8)	2 (4.5)	10 (9.1)	0.54	40 (18.0)	16 (16.3)	24 (19.4)	0.56
Others	11 (7.1)	2 (4.5)	9 (8.2)	0.66	18 (8.1)	10 (10.2)	8 (6.5)	0.31
Disease severity at defined time^b^								
MAP^c^, mean ± SD	82.2 ± 10.0	80.3 ± 7.4	83.0 ± 10.8	0.08	64.2 ± 12.2	63.1 ± 11.0	65.0 ± 13.1	0.25
APACHE II score, median (IQR)	15 (11–19)	15 (12–22)	15 (11–18)	0.44	15 (10–20)	16 (13–20)	14 (10–19)	0.25
SOFA score, median (IQR)	7 (4–9)	6 (4–9)	7 (5–9)	0.14	7 (5–10)	7 (5–10)	7 (5–10)	0.78
NRS score, median (IQR)	4 (3–4)	3 (3–4)	4 (3–4)	0.76	3 (3–4)	4 (3–4)	3 (3–4)	0.31
mNUTRIC score, median (IQR)	4 (3–5)	4 (3–6)	4 (3–5)	0.58	4 (3–5)	4 (3–5)	4 (2–5)	0.60
Mechanical ventilation, *n* (%)	99 (64.3)	32 (72.7)	67 (60.9)	0.17	176 (79.3)	73 (74.5)	103 (83.1)	0.12
AGI grade II, n (%)	71 (46.1)	21 (47.7)	50 (45.5)	0.80	107 (48.2)	52 (53.1)	55 (44.4)	0.20
Primary AGI^a^, *n* (%)	18 (11.7)	6 (13.6)	12 (10.9)	0.63	86 (38.7)	44 (44.9)	42 (33.9)	0.09
ICU course prior to defined time^b^								
Serum albumin, mean ± SD, g/L	30.2 ± 6.7	31.4 ± 7.3	29.7 ± 6.5	0.17	26.9 ± 7.2	27.0 ± 7.4	26.9 ± 7.0	0.87
Feeding route, *n* (%)				0.14				0.67
Feeding via nasogastric tube	141 (91.6)	38 (86.4)	103 (93.6)		210 (94.6)	92 (93.9)	118 (95.2)	
Feeding via nasojejunal tube	13 (8.4)	6 (13.6)	7 (6.4)		12 (5.4)	6 (6.1)	6 (4.8)	
EN calorie intake on the 3th day, median (IQR), (kcal)	864 (480–1,440)	864 (480–1,440)	864 (432–1,440)	0.28	0 (0–0)	0 (0–0)	0 (0–0)	0.15
EN protein intake on the 3th day, median (IQR), (g)	34.5 (19.2–57.6)	32.6 (19.2–48.0)	38.4 (19.2–57.6)	0.30	0 (0–0)	0 (0–0)	0 (0–0)	0.04
EN calorie intake on the 7th day, median (IQR), (kcal)	1,080 (720–1728)	1,560 (1080–1800)	1,080 (720–1,440)	0.001	960 (480–1,440)	960 (480–1,340)	1,080 (720–1,440)	0.02
EN protein intake on the 7th day, median (IQR), (g)	43.2 (28.8–65.3)	61.4 (40.8–72)	43.2 (28.8–57.6)	0.002	38.4 (24–57.6)	38.4 (19.2–57.6)	45.6 (28.8–57.6)	0.01
Supplemental parenteral nutrition use, *n* (%)	13 (8.4)	3 (6.8)	10 (9.1)	0.759	27 (12.2)	15 (15.3)	12 (9.7)	0.22

ICU, intensive care unit; BMI, body mass index; MAP, mean arterial pressure; APACHE, Acute Physiology, and Chronic Health Evaluation; SOFA, Sequential Organ Failure Assessment; NRS, nutrition risk screening 2002 score; mNUTRIC, modified NUTRIC score; AGI, acute gastrointestinal injury, EN, enteral nutrition; IQR, interquartile range.

^a^Primary AGI (2) is associated with primary disease or direct injury to organs of the GI system (first hit); ^b^The defined time period was the first 24 h of ICU admission; ^c^MAP represents the worst mean arterial pressure before application of vasoactive drugs during ICU admission.

### 3.2 Primary outcomes

#### 3.2.1 Caloric adequacy

**Figure 2:**
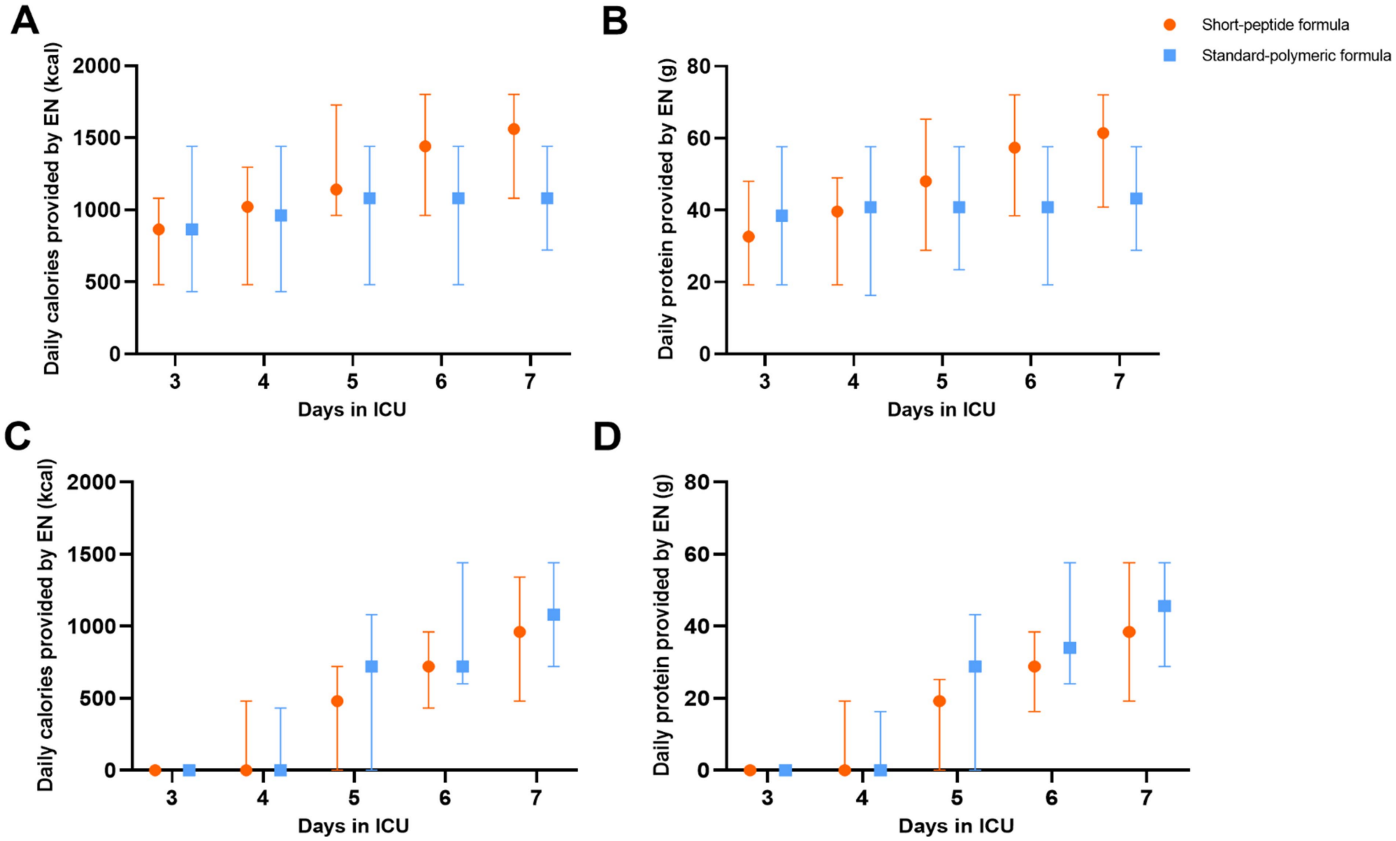
In the EEN **(A,B)** and DEN **(C,D)** subgroups, daily calories and protein provided by EN in the short-peptide group and the standard-polymer group.

In the EEN subgroup, the caloric adequacy of EN on day 7 in the short-peptide group was higher than that of the standard-polymeric group (59.1% vs. 27.3%, *p* = 0.001). However, in the DEN subgroup, the caloric adequacy of EN on day 7 in the short-peptide group was lower than that of the standard-polymeric group (28.6% vs. 43.5%, *p* = 0.02) (Table 3 and Figure 2).

**Table 3:** Comparison of nutritional outcomes and adverse gastrointestinal events.

Nutrition summary	EEN subgroup				DEN subgroup			
	Short-peptide (*n* = 44)	Standard-polymeric (*n* = 110)	*χ* ^2^ */Z*	*P-*value	Short-peptide (*n* = 98)	Standard-polymeric (*n* = 124)	*χ* ^2^ */Z*	*P-*value
Timing of EN, median (IQR), hours	14.5 (3, 21)	14 (2, 24)	−0.48	0.63	94 (79.8, 117.3)	96 (72, 118)	−0.30	0.77
Caloric adequacy of EN on the 7th day^a^, median (IQR), (%)	26 (59.1)	30 (27.3)	13.75	0.001**	28 (28.6)	54 (43.5)	5.27	0.02*
Calories gain from EN within 3–7d, median (IQR), g	624 (0, 936)	0 (−360, 480)	−3.55	0.001**	960 (480, 1,200)	1,080 (720, 1,440)	−2.10	0.04*
Protein gain from EN within 3–7d, median (IQR), kcal	24 (0, 38)	0 (−14.4, 19.2)	−2.96	0.003**	38.4 (19.2, 50.4)	43.2 (28.8, 57.6)	−2.10	0.04*
Gastric retention (%)	8 (18.2)	40 (36.4)	4.84	0.03*	14 (14.3)	26 (21.0)	1.66	0.20
Diarrhea (%)	4 (9.1)	28 (25.5)	5.11	0.02*	10 (10.2)	20 (16.1)	1.64	0.20

EN, enteral nutrition; IQR, interquartile range.

^a^In this study, we defined the caloric adequacy of EN as >80% of the caloric target; *p < 0.05, **p < 0.01.

Supplementary Tables S1, S2 describe the results of the logistic regression analysis for the EEN and DEN subgroups, respectively. mNUTRIC and APACHE II scores were excluded from the multivariate analysis because of their high collinearity (Supplementary Figures S2, S3). The short-peptide formulas were associated with EN caloric adequacy on day 7 after controlling for covariates in the EEN subgroup (*OR:* 3.852, 95% *CI:* 1.851–8.017; *p* < 0.001). In addition, in the DEN subgroup, the EN caloric adequacy on day 7 associated with the standard-polymeric formulas (*OR:* 2.409, 95% *CI:* 1.325–4.378; *p =* 0.004), gastric retention (*OR:* 0.272, 95% *CI:* 0.115–0.643; *p* = 0.003), and diarrhea (*OR:* 0.548, 95% *CI:* 0.378–0.796; *p* = 0.002).

### 3.3 Secondary outcomes

#### 3.3.1 Calories and protein gain

In the EEN subgroup, calories and protein gain from EN were higher in the short-peptide group than in the standard-polymeric group (624[0, 936] vs. 0[− 360, 480], *p* = 0.001; 24[0, 38] vs. 0[− 14.4, 19.2], *p* = 0.003, respectively). However, in the DEN subgroup, calories and protein gain from EN were lower in the short-peptide group than in the standard-polymeric group (960[480, 1,200] vs. 1,080[720, 1,440], *p* = 0.04; 38.4[19.2, 50.4] vs. 43.2[28.8, 57.6], *p* = 0.04, respectively) (Table 3 and Figure 2). Associations between calories/protein gain from EN within 3-7d and critical illness and clinical outcomes are presented in Supplementary Table S4.

#### 3.3.2 Adverse gastrointestinal events

**Figure 3:**
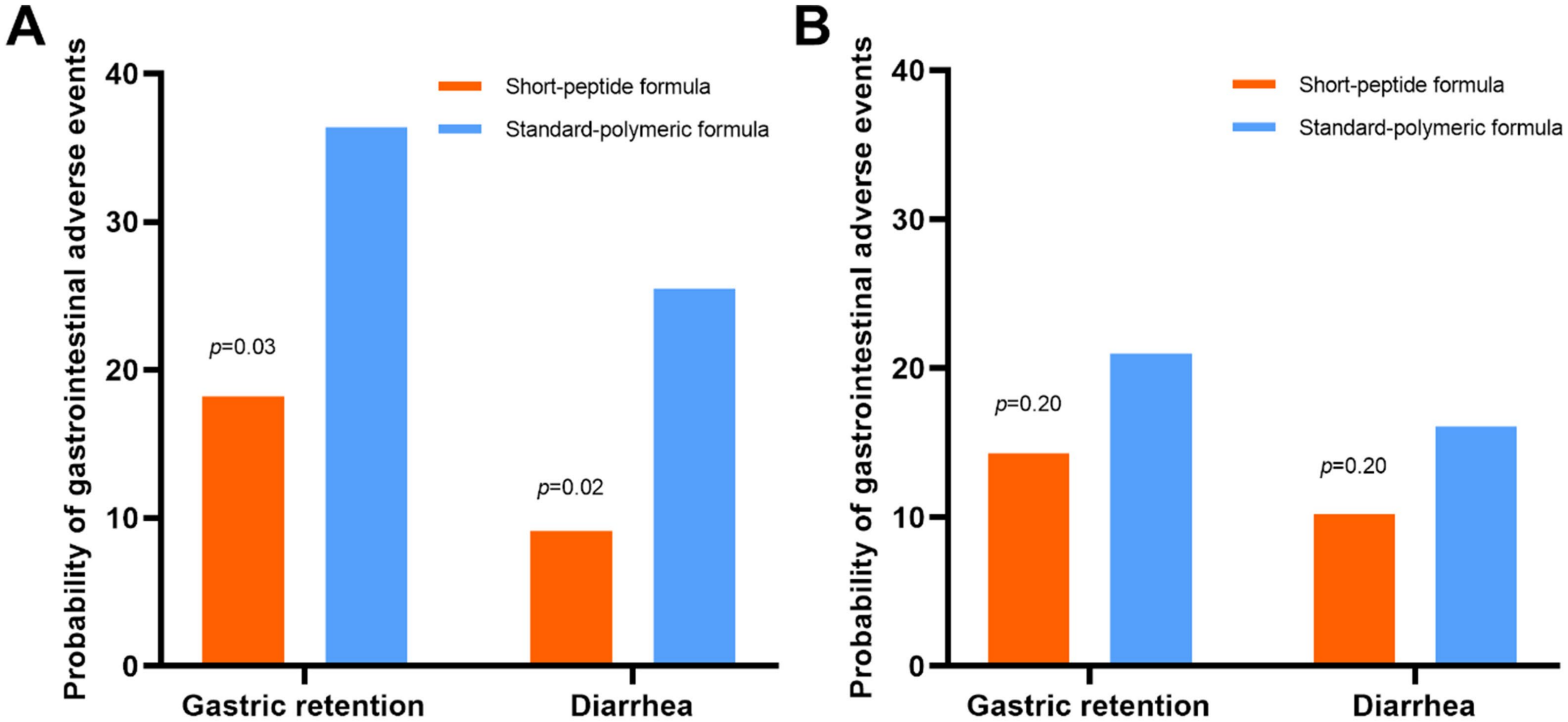
In the EEN **(A)** and DEN **(B)** subgroups, the incidence of gastric retention and diarrhea in the short-peptide group and the standard-polymer group.

In the EEN subgroup, the incidence of gastric retention (18.2% vs. 36.4%, *p* = 0.03) and diarrhea (9.1% vs. 25.5%, *p* = 0.02) was lower in the short-peptide group than in the standard-polymeric group. However, in the DEN subgroup, there were no statistically significant differences in the incidence of gastric retention and diarrhea between the short peptide and standard polymeric groups (*p* > 0.05) (Table 3 and Figure 3).

#### 3.3.3 Daily caloric EN/(EN + PN) ratios

**Figure 4:**
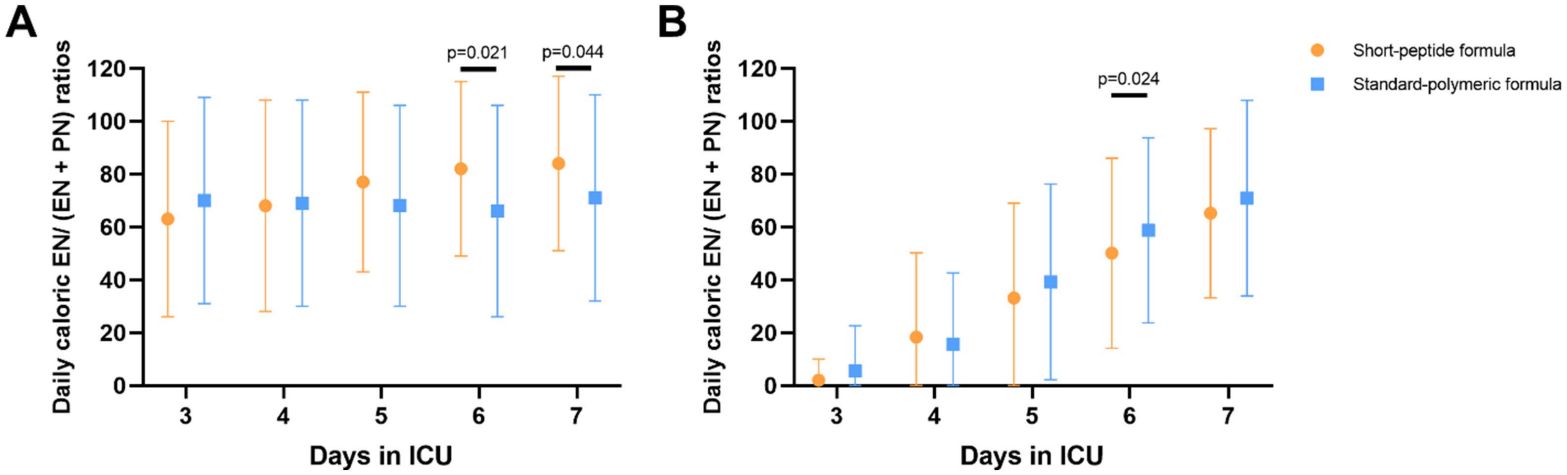
In the EEN **(A)** and DEN **(B)** subgroups, comparison of daily caloric EN/(EN + PN) ratios on 3–7 days after ICU admission. Statistical comparisons between the two groups shown in the figure were conducted using the Mann–Whitney *U* test.

In the EEN subgroup, caloric EN/(EN + PN) ratios were higher on day 6 and 7 in the short-peptide group than in the standard-polymeric group (*p* = 0.021; *p* = 0.044, respectively). In the DEN subgroup, caloric EN/(EN + PN) ratios were higher on day 6 in the standard-polymeric group than in the short-peptide group (*p* = 0.024) (Supplementary Table S5 and Figure 4).

#### 3.3.4 Clinical outcomes

**Figure 5:**
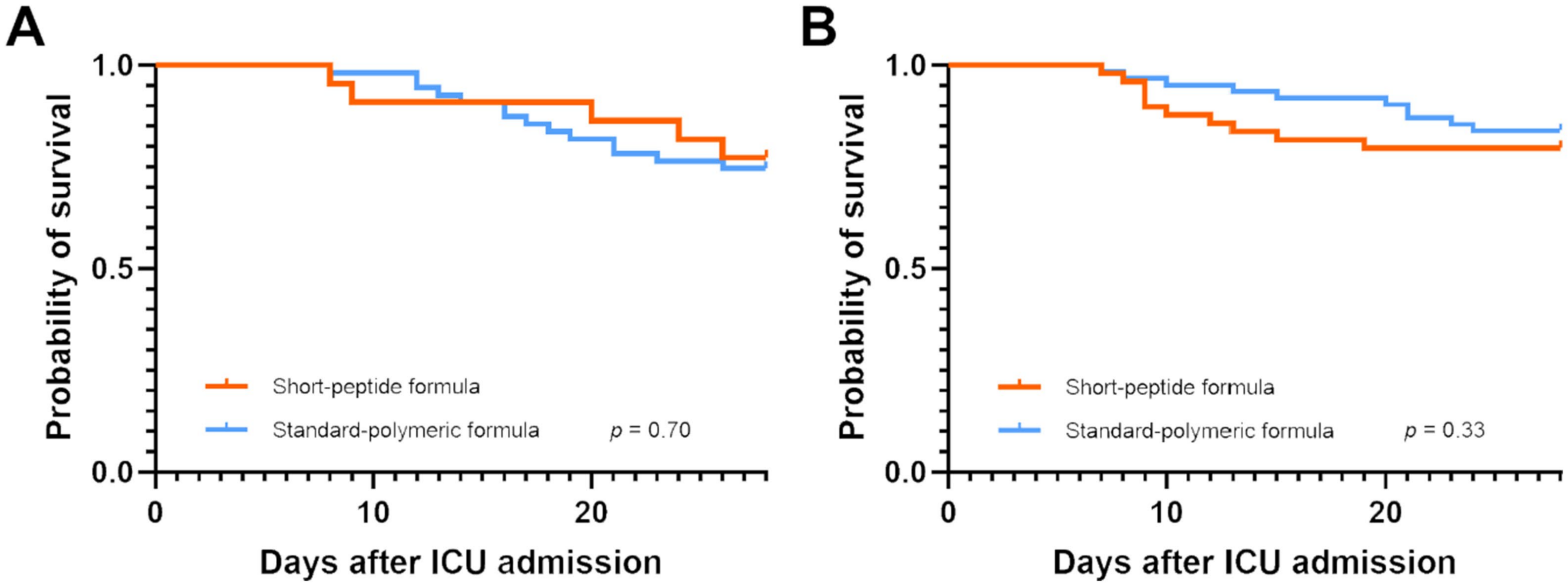
Kaplan–Meier survival curves for patients with AGI grade I both in the EEN **(A)** and DEN **(B)** subgroup.

There were no statistically significant differences were observed in ICU and 28-day mortality, ICU length of stay, hospital length of stay, ventilator-free days and hospitalization cost in the short-peptide and standard-polymeric formulas group in both the EEN and DEN subgroups (*p* > 0.05) (Supplementary Table S6). Kaplan–Meier survival curves plotted with the 28-day mortality rates also did not show a statistically significant difference between the EEN (*log-rank p* = 0.70) and DEN subgroups (*log-rank p* = 0.33) (Figure 5). In Supplementary Table S7, we report the uncorrected and FDR-corrected *p*-values for all secondary outcomes to address potential issues related to multiple comparisons.

## 4 Discussion

This study compared the effects of different EN formulas on clinical outcomes in critically ill sepsis patients with AGI grades I-II, with subgroup analysis based on the timing of EN initiation (EEN and DEN subgroups). In the EEN subgroup, the short-peptide formula resulted in higher caloric adequacy, as well as greater calorie and protein gain from EN compared to the standard-polymer group. It also showed lower incidence of gastric retention and diarrhea, along with a higher EN/(EN + PN) ratio on days 6 and 7. However, in the DEN subgroup, the standard-polymer formula had higher caloric adequacy and calorie/protein gain compared to the short-peptide formula. There were no significant differences in gastric retention or diarrhea between the two groups, but the standard-polymer group had a higher EN/(EN + PN) ratio on day 3. Both formulas showed similar outcomes in terms of ICU and hospital stay, costs, mortality, and ventilator-free days.

The EEN may be beneficial for patients with sepsis and AGI (it can maintain gut integrity and prevent intestinal permeability) (18, 21); however, it may also lead to intestinal ischemia and gastrointestinal adverse events, which can lead to feeding intolerance syndrome and a more severe gastrointestinal injury. The short-peptide EN formulas can be absorbed without digestive enzymes, which may reduce the incidence of intolerance and undesired gastrointestinal events in patients (19). The short-peptide EN formulas can be digested and absorbed faster from the gut, make amino acids more available after meals, and promote the binding of dietary amino acids to skeletal muscle protein (24). It has been shown that *N*-formyl-methionyl-leucyl-phenylalanine transport in the colon of rats increases the expression of oligopeptide transporter, which may damage colonic mucosa (25, 26). Short-peptides can protect the intestinal mucosa and reduce intestinal damage because they can competitively inhibit *N*-formyl-methionyl-leucyl-phenylalanine transport or have a greater transport efficiency (27). In the early acute phase of critical illness (≤48 h) (9), the short-peptide formulas may be more suitable for critically ill patients with sepsis and AGI grade I than the standard-polymeric formulas. The feeding tolerance outcomes and nutritional adequacy outcomes in the EEN group support this view. Furthermore, the findings related to calorie and protein gain from EN within 3–7d remained statistically robust even after applying FDR correction. These results highlight the potential clinical significance of the observed effects and underscore the need for further validation in larger, prospective studies. Studies have showed (28), the higher the average EN/(EN + PN) ratio of patients admitted to ICU within 7 days, the lower the in-hospital mortality. In the EEN subgroup, the short-peptide formula group had a higher EN proportion on days 6 and 7, which may indicate some potential benefits. However, no differences were found in other clinical outcomes between the two groups. These results are similar to those of previous studies (19, 29–33). According to our results, the short-peptide formulas can be considered in critically ill patients with sepsis and AGI grade I or II, if the administration of EN is started within 48 h.

The DEN subgroup had a lower MAP (72.4 mmHg vs. 86.7 mmHg) and a higher percentage of primary AGI (38.7% vs. 11.7%) than the EEN subgroup. The gastrointestinal tract of patients with primary AGI may require more rest than that of patients with secondary AGI, and patients with lower MAP are at a higher risk of intestinal ischemia. We had proposed the gut rest strategy (trophic feeding after 72 h of ICU admission may be the better option for AGI patients) (34). Therefore, it is reasonable to delay the EN start timing. In the DEN subgroup of this study, the caloric adequacy of EN on day 7, and calories and protein gain from EN within 3–d in the standard-polymeric group was higher. This unexpected result seems difficult to explain, especially since the incidence of gastric retention and diarrhea was similar between the two groups. One possible reason for this outcome could be the difference in energy density between the two EN formulas (short-peptide formula 1.0 kcaL/mL vs. standard polymer formula 1.5 kcaL/mL), making it easier for the standard polymer group to achieve nutritional goals. In the late acute phase of critical illness (˃48 h) (9), metabolic function and gastrointestinal digestion and absorption function were gradually recovered in critically ill patients. The gastrointestinal blood flow and permeability were improved, the digestive system had a good rest (34), and the tolerance of the gastrointestinal tract might be stronger. In this case, the disadvantage of short peptides is magnified, and this advantage is difficult to achieve. Therefore, the standard-polymeric formulas may be considered for critically ill patients with sepsis and AGI grades I–II if EN administration starts after 48 h. Unfortunately, in the DEN group, there was no statistical difference in all secondary outcomes after FDR adjustment.

An interesting problem was found in this study: in the DEN subgroup, the standard-polymeric formulas was associated with caloric adequacy of EN on day 7; however, diarrhea and gastric retention were also associated with EN caloric adequacy on day 7. This is because, based on late initiation of EN, it is more difficult to reach caloric adequacy of EN on day 7 if diarrhea and gastric retention occur. The relatively small sample size of the short-peptide group in the EEN subgroup may represent an interesting issue. One possible explanation for this observation is that clinicians who prefer short-peptide formulas may be more inclined to delay EN initiation when managing sepsis patients with AGI, potentially due to concerns about gastrointestinal tolerance or other clinical considerations. In addition, in the correlation analysis of the calories/protein gain from EN within 3-7d with critical illness and clinical outcomes, it can be found that the nutritional increase in the EEN group is associated with the nutritional formula and APACHE II score > 15, while in the DEN group it is associated with MAP and gastrointestinal symptoms. It may be indicated that there are differences between the EEN group and the DEN group. This exploratory result requires careful interpretation.

This study had some limitations. First, this study is retrospective in design and therefore inherently subject to selection bias. The clinicians’ choice of nutritional formulas represents the most significant source of such bias. Additionally, the imbalance between groups could have contributed to residual bias, leading to a selection bias. Although baseline characteristics were relatively balanced between the two formula groups across subgroups, it should be acknowledged that the short-peptide group exhibited higher rates of nasojejunal tube utilization and a greater proportion of primary AGI cases. These limitations are inherent to retrospective studies and underscore the need for prospective investigations to validate our findings. Second, the caloric target was calculated with an equation rather than indirect calorimetry, which may affect the accuracy of the caloric goal calculation. Third, we were unable to draw evidence-based conclusions regarding when EN should be initiated for patients with sepsis and AGI grades I–II because of uneven baselines in the two parts (the provision of EN within 48 h and the provision of EN after 48 h). Fourth, this study only included patients with AGI grades I–II, which would limit the extrapolation of these exploratory findings. Finally, the relatively small and unbalanced sample size, especially in the EEN subgroup, which may affect the generalizability and reliability of the results. Although the sample size exceeded the calculated requirement, it must be acknowledged that these exploratory results may lack robustness. However, this was the first study to explore the impact of short-peptide and standard-polymeric EN formulas on feeding and clinical outcomes on severe sepsis patients with AGI. Further randomized control trials are required to confirm the results of our study.

## 5 Conclusion

In this retrospective exploratory cohort of sepsis patients with AGI grades I-II, preliminary findings observed that short-peptide formulas may be considered for those who initiate EN early (≤48 h), while standard-polymer formulas may be an option for those who initiate EN late (>48 h). These observations did not translate into differences in hard outcomes and should be regarded as hypothesis-generating. Prospective, adequately powered studies are needed before any practice implications can be drawn.

## Data Availability

The raw data supporting the conclusions of this article will be made available by the authors, without undue reservation.
