# Supplementary material for: Comparison of standard polymer formula versus short peptide formula in sepsis patients with acute gastrointestinal injury
Source: Front Nutr. 2025 Oct 20;12:1682020. doi: 10.3389/fnut.2025.1682020 (PMC12580080; doi:10.3389/fnut.2025.1682020)
Supplement: Supplementary file 1 [file Table_1.DOCX]

**Supplementary Information**

Supplementary material Figure S1: EN startup ratios of EEN (A) and DEN (B) subgroup

Supplementary material Figure S2: Correlation of the variables in multivariate analysis (EEN subgroup)

Supplementary material Figure S3: Correlation of the variables in multivariate analysis (DEN subgroup)

Supplementary material Table S1: The comparison of the characteristics of two nutritional formulas

Supplementary material Table S2: Multivariate predictors of EN Caloric adequacy on the 7th day (EEN group)

Supplementary material Table S3: Multivariate predictors of EN Caloric adequacy on the 7th day (DEN group)

Supplementary material Table S4: Associations between calories/protein gain from EN within 3-7d and critical illness and clinical outcomes.

Supplementary material Table S5 Comparison of daily caloric EN/ (EN + PN) ratios on 3-7 days after ICU admission

Supplementary material Table S6: Comparison of clinical outcomes

Supplementary material Table S7: Uncorrected and FDR-Corrected p-Values for Multiple Comparisons


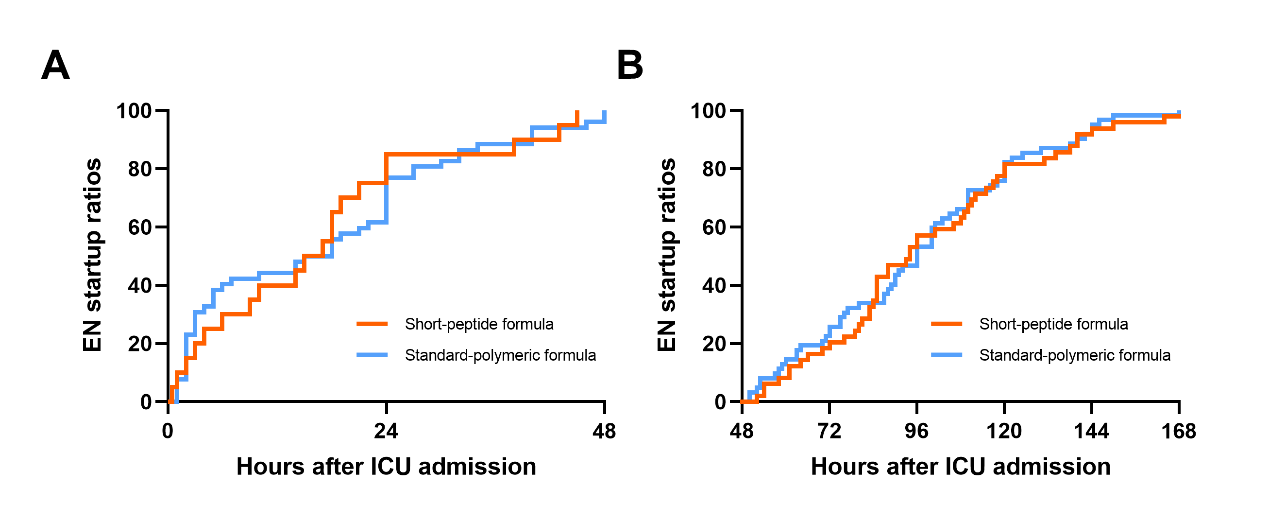


**Figure S**1. EN startup ratios of EEN (A) and DEN (B) subgroup. EN, Enteral nutrition; ICU, Intensive care unit.


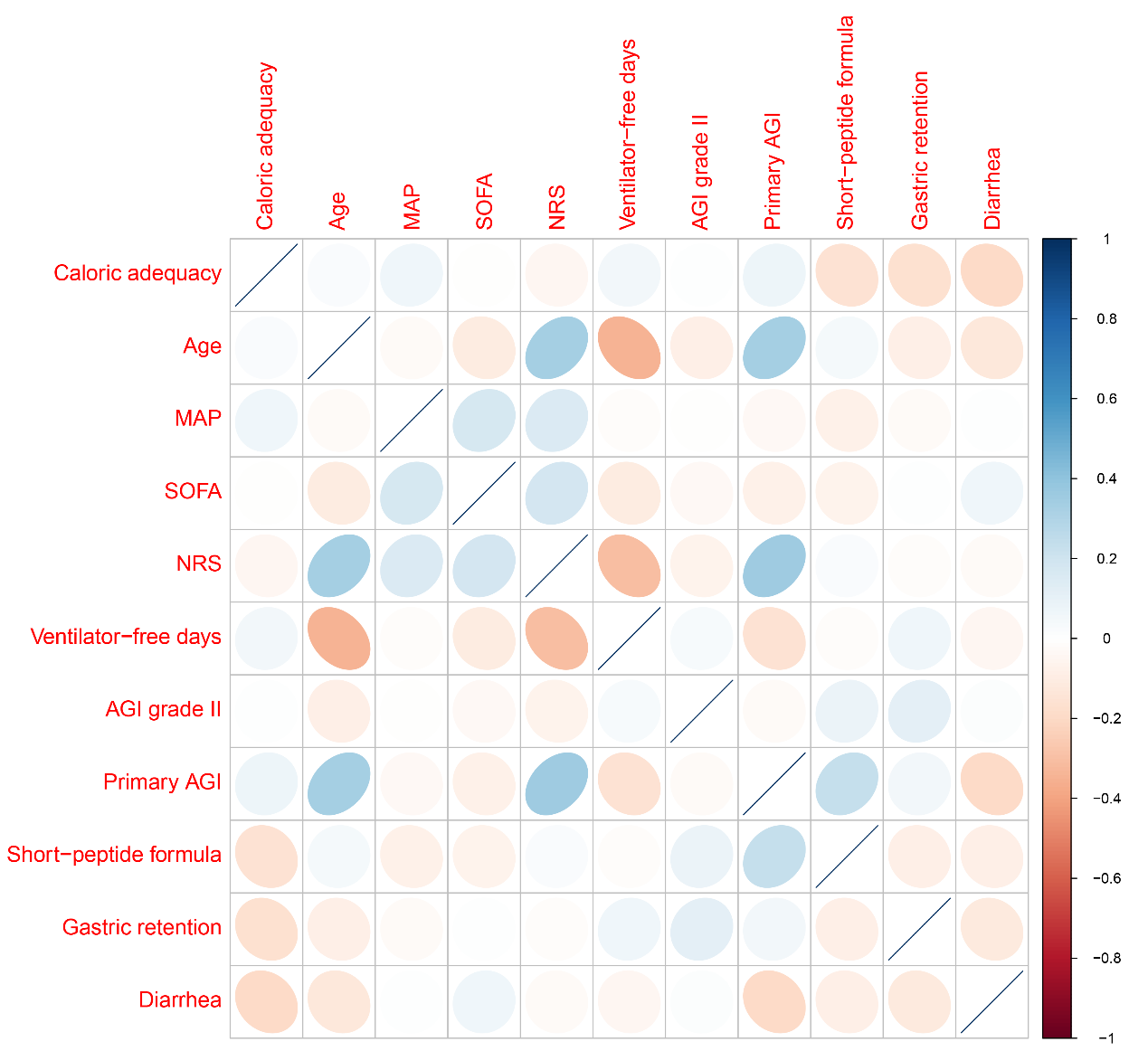


**Figure S2.** Correlation of the variables in multivariate analysis (EEN subgroup). MAP, Mean arterial pressure; SOFA, Sequential Organ Failure Assessment; NRS, nutrition risk screening 2002 score; AGI, acute gastrointestinal injury.


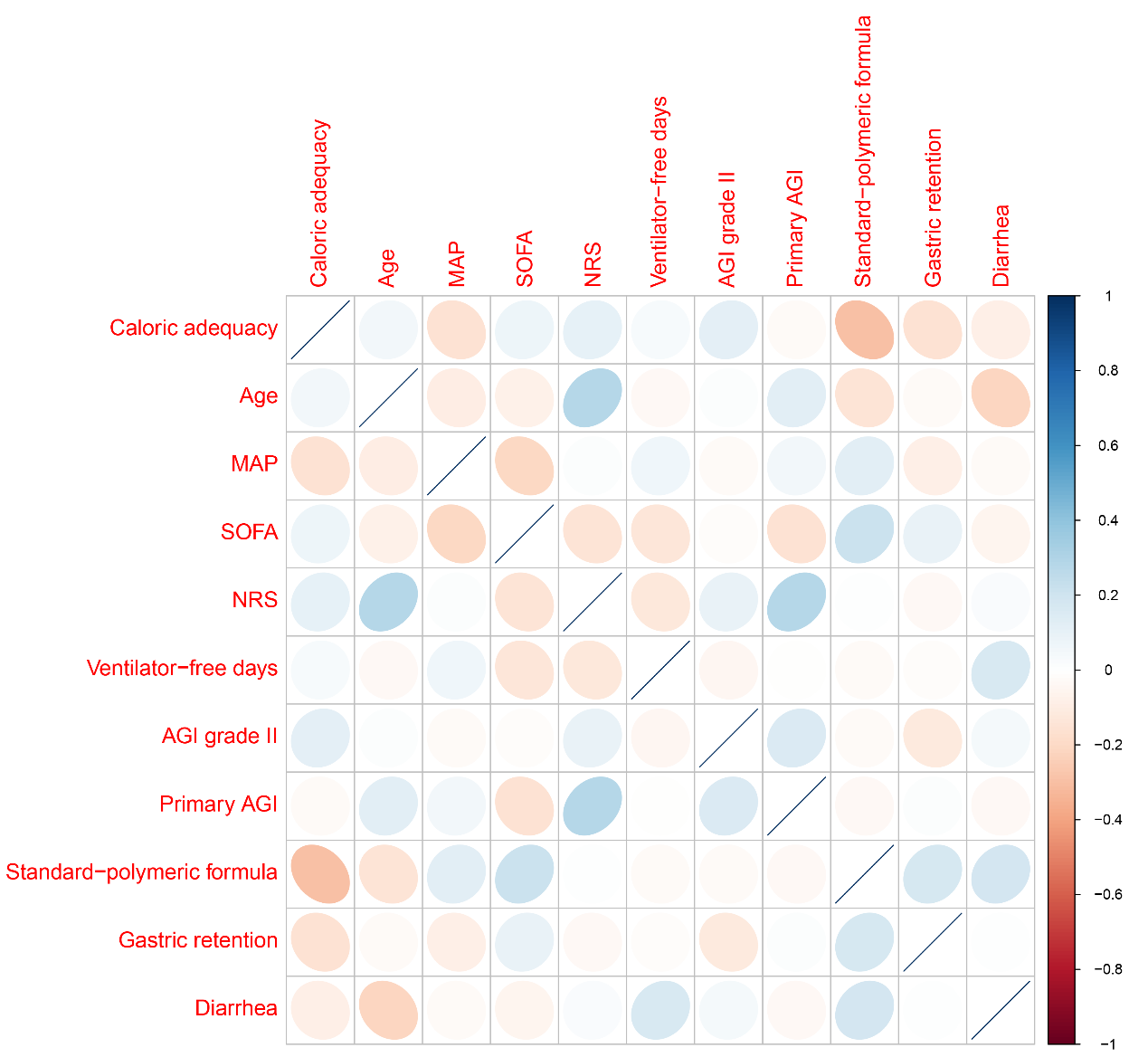


**Figure S3.** Correlation of the variables in multivariate analysis (DEN subgroup). MAP, Mean arterial pressure; SOFA, Sequential Organ Failure Assessment; NRS, nutrition risk screening 2002 score; AGI, acute gastrointestinal injury.

**Table S1**: The comparison of the characteristics of two nutritional formulas

| Characteristics | Standard Polymer Formula | Short Peptide Formula |
| --- | --- | --- |
| Calorie density | 750kcal/500ml | 500kcal/500ml |
| Osmotic pressure | 300mOsm/L | 440mOsm/L |
| Protein | 30g/500ml, casein as the main component | 20g/500ml, whey protein is the main component |
| Carbohydrates | 92.5g/500ml | 88g/500ml |
| Fat | 29.2g/500ml, MCT: 15.5% | 8.5g/500ml, MCT: 60.5% |
| Dietary fiber | Yes | No |

Abbreviations: MCT, Medium-Chain Triglycerides.

**Table S2.** Multivariate predictors of EN Caloric adequacy on the 7th day (EEN group).

| Variable | Univariate | | |  | Multivariate | | |
| --- | --- | --- | --- | --- | --- | --- | --- |
|  | *OR* | (95%*CI)* | *P Value* |  | *OR* | (95%*CI)* | *P Value* |
| Age | 0.987 | (0.956 - 1.020) | 0.44 |  |  |  |  |
| MAP ^a^ | 0.969 | (0.929 - 1.010) | 0.14 |  |  |  |  |
| SOFA | 1.066 | (0.917 - 1.238) | 0.41 |  |  |  |  |
| NRS | 1.660 | (1.051 - 2.622) | 0.03* |  | 1.268 | (0.883 - 1.821) | 0.20 |
| Ventilator - free days | 1.021 | (0.983 - 1.061) | 0.28 |  |  |  |  |
| AGI grade II | 0.504 | (0.228 - 1.111) | 0.09 |  | 0.601 | (0.291 - 1.239) | 0.17 |
| Primary AGI | 0.659 | (0.189 - 2.300) | 0.51 |  |  |  |  |
| Short-peptide formula | 5.728 | (2.246 - 14.611) | < 0.001** |  | 4.197 | (1.974 - 8.924) | < 0.001** |
| Gastric retention | 0.400 | (0.153 - 1.043) | 0.06 |  | 0.544 | (0.241 - 1.230) | 0.14 |
| Diarrhea | 0.963 | (0.326 - 2.845) | 0.95 |  |  |  |  |
| Underlying conditions |  |  |  |  |  |  |  |
| Hypertension | 1.820 | (0.714 - 4.640) | 0.21 |  |  |  |  |
| Chronic obstructive pulmonary disease | 2.736 | (0.898 - 8.336) | 0.08 |  | 2.362 | (0.926 - 6.026) | 0.07 |
| Coronary heart disease | 1.335 | (0.503 - 3.544) | 0.56 |  |  |  |  |
| Diabetes mellitus | 1.664 | (0.680 - 4.071) | 0.26 |  |  |  |  |

Abbreviations: EN, enteral nutrition; ICU, intensive care unit; SOFA, Sequential Organ Failure Assessment; NRS, Nutrition Risk Screening 2002 score; AGI, acute gastrointestinal injury; **p*< 0.05, ***p*< 0.01.

^a^ MAP represents the worst mean arterial pressure before the application of vasoactive drugs during ICU admission.

**Table S3.** Multivariate predictors of EN Caloric adequacy on the 7th day (DEN group).

| Variable | Univariate | | |  | Multivariate | | |
| --- | --- | --- | --- | --- | --- | --- | --- |
|  | *OR* | (95%*CI)* | *P Value* |  | *OR* | (95%*CI)* | *P Value* |
| Age | 0.992 | (0.969 - 1.016) | 0.52 |  |  |  |  |
| MAP ^a^ | 1.014 | (0.988 - 1.041) | 0.30 |  |  |  |  |
| SOFA | 0.968 | (0.872 - 1.074) | 0.54 |  |  |  |  |
| NRS | 0.853 | (0.653 - 1.114) | 0.24 |  |  |  |  |
| Ventilator-free days | 1.014 | (0.983 - 1.046) | 0.39 |  |  |  |  |
| AGI grade II | 0.813 | (0.439 - 1.504) | 0.51 |  |  |  |  |
| Primary AGI | 2.120 | (1.030 - 4.364) | 0.04* |  | 1.482 | (0.809 - 2.713) | 0.20 |
| Standard-polymeric formula | 3.096 | (1.597 - 6.002) | 0.001** |  | 2.409 | (1.325 - 4.378) | 0.004** |
| Gastric retention | 0.120 | (0.041 - 0.351) | 0.001** |  | 0.272 | (0.115 - 0.643) | 0.003** |
| Diarrhea | 0.110 | (0.029 - 0.423) | 0.001** |  | 0.165 | (0.054 - 0.504) | 0.002** |
| Underlying conditions |  |  |  |  |  |  |  |
| Hypertension | 0.885 | (0.466 - 1.680) | 0.71 |  |  |  |  |
| Chronic obstructive pulmonary disease | 1.286 | (0.639 - 2.589) | 0.48 |  |  |  |  |
| Coronary heart disease | 0.957 | (0.484 - 1.891) | 0.90 |  |  |  |  |
| Diabetes mellitus | 1.148 | (0.606 - 2.175) | 0.67 |  |  |  |  |

Abbreviations: EN, enteral nutrition; ICU, intensive care unit; APACHE, Acute Physiology and Chronic Health Evaluation; SOFA, Sequential Organ Failure Assessment; NRS, Nutrition Risk Screening 2002 score; mNUTRIC, modified NUTRIC score; AGI, acute gastrointestinal injury; **p*< 0.05, ***p*< 0.01.

^a^ MAP represents the worst mean arterial pressure before the application of vasoactive drugs during ICU admission.

**Table S4**. Associations between calories/protein gain from EN within 3-7d and critical illness and clinical outcomes.

|  | EEN subgroup | | | |  | DEN subgroup | | | |
| --- | --- | --- | --- | --- | --- | --- | --- | --- | --- |
|  | Calories | *P Value* | Protein | *P Value* |  | Calories | *P Value* | Protein | *P Value* |
| EN formulas | -0.287 | < 0.001* | -0.240 | 0.003* |  | 0.139 | 0.039 | 0.134 | 0.047 |
| APACHE II score > 15 | -0.169 | 0.036* | -0.141 | 0.080 |  | -0.09 | 0.183 | -0.08 | 0.236 |
| MAP | 0.081 | 0.319 | 0.069 | 0.397 |  | -0.254 | < 0.001* | -0.253 | < 0.001* |
| Gastric retention | -0.129 | 0.111 | -0.109 | 0.178 |  | -0.247 | < 0.001* | -0.255 | < 0.001* |
| Diarrhea | -0.023 | 0.778 | -0.031 | 0.702 |  | -0.07 | 0.298 | -0.104 | 0.123 |
| ICU mortality | -0.098 | 0.228 | -0.087 | 0.285 |  | -0.017 | 0.797 | -0.046 | 0.495 |
| 28 - day mortality | -0.106 | 0.189 | -0.1 | 0.217 |  | -0.099 | 0.140 | -0.062 | 0.357 |

Abbreviations: EN, enteral nutrition; MAP, mean arterial pressure; **p*< 0.05.

**Table S5.** Comparison of daily caloric EN/ (EN + PN) ratios on 3-7 days after ICU admission.

| **N**utrition Summary | EEN subgroup | | | |  | DEN subgroup | | | |
| --- | --- | --- | --- | --- | --- | --- | --- | --- | --- |
|  | Short-peptide (n = 44) | Standard-polymeric  (n = 110) | *Z* | *P Value* |  | Short-peptide (n = 98) | Standard-polymeric  (n = 124) | *Z* | *P Value* |
| EN/ (EN + PN) ratios on the 3rd day, median (IQR), (%) | 62 (34 – 100) | 100 (39 – 100) | -1.259 | 0.208 |  | 0 (0 – 0) | 0 (0 – 0) | -1.424 | 0.155 |
| EN/ (EN + PN) ratios on the 4th day, median (IQR), (%) | 100 (34 – 100) | 100 (32 – 100) | -0.078 | 0.938 |  | 0 (0 – 32) | 0 (0 – 28) | -0.228 | 0.820 |
| EN/ (EN + PN) ratios on the 5th day, median (IQR), (%) | 100 (50 – 100) | 100 (34 – 100) | -1.435 | 0.151 |  | 25 (0 – 48) | 38 (0 – 60) | -1.405 | 0.160 |
| EN/ (EN + PN) ratios on the 6th day, median (IQR), (%) | 100 (76 – 100) | 100 (34 – 100) | -2.312 | 0.021* |  | 38 (25 – 100) | 53 (33 – 100) | -2.258 | 0.024* |
| EN/ (EN + PN) ratios on the 7th day, median (IQR), (%) | 100 (100 – 100) | 100 (40 – 100) | -2.011 | 0.044* |  | 55 (34 – 100) | 100 (42 – 100) | -1.511 | 0.131 |

Abbreviations: EN, enteral nutrition; PN, parenteral nutrition; **p*< 0.05.

**Table S6.** Comparison of clinical outcomes.

| Clinical outcomes | EEN subgroup | | | |  | DEN subgroup | | | |
| --- | --- | --- | --- | --- | --- | --- | --- | --- | --- |
|  | Short-peptide (n = 44) | Standard-polymeric (n = 110) | *χ²/Z* | *P Value* |  | Short-peptide (n = 98) | Standard-polymeric (n = 124) | *χ²/Z* | *P Value* |
| ICU Length of stay, median (IQR), day | 14 (11,25) | 15 (10,25) | - 0.01 | 0.99 |  | 14 (9,20) | 12 (9,20) | - 1.43 | 0.15 |
| Hospital length of stay, median (IQR), day | 24 (13,39) | 25 (14,34) | - 0.51 | 0.61 |  | 18 (12,25) | 19 (13,28) | - 0.72 | 0.47 |
| Hospitalization cost, median (IQR), ten thousand yuan | 10.8 (6.7,18.2) | 12.1 (8.3,20.4) | - 1.09 | 0.28 |  | 15.5 (7.0,25.3) | 10.3 (6.5,20.9) | -1.136 | 0.26 |
| ICU mortality (%) | 6 (13.6) | 22 (20.0) | 0.86 | 0.36 |  | 14 (14.3) | 10 (8.1) | 2.2 | 0.14 |
| 28 - day mortality (%) | 10 (22.7) | 28 (25.5) | 0.13 | 0.72 |  | 20 (20.4) | 20 (16.1) | 0.68 | 0.41 |
| Ventilator - free days, median (IQR), day | 8 (2,17) | 8 (3,15) | 0.01 | 1.0 |  | 7 (2,13) | 8 (4,13) | - 1.36 | 0.17 |

Abbreviations: ICU, intensive care unit; IQR, interquartile range.

**Table S7. Uncorrected and FDR-Corrected p-Values for Multiple Comparisons**

| Outcomes | EEN subgroup | |  | DEN subgroup | |
| --- | --- | --- | --- | --- | --- |
|  | *P Value* | *FDR-corrected P-value* |  | *P Value* | *FDR-corrected P-value* |
| Calories gain from EN within 3-7d | 0.001** | 0.013* |  | 0.04* | 0.116 |
| Protein gain from EN within 3-7d | 0.003** | 0.026* |  | 0.04* | 0.104 |
| Gastric retention | 0.03* | 0.098 |  | 0.20 | 0.325 |
| Diarrhea | 0.02* | 0.104 |  | 0.20 | 0.306 |
| Caloric EN/ (EN + PN) ratios on the 6th day | 0.021 | 0.091 |  | 0.024 | 0.089 |
| Caloric EN/ (EN + PN) ratios on the 7th day | 0.044 | 0.104 |  | 0.131 | 0.28 |
| ICU Length of stay | 0.99 | 1.0 |  | 0.15 | 0.279 |
| Hospital length of stay | 0.61 | 0.690 |  | 0.47 | 0.555 |
| Hospitalization cost | 0.28 | 0.383 |  | 0.26 | 0.376 |
| ICU mortality | 0.36 | 0.468 |  | 0.14 | 0.280 |
| 28-day mortality | 0.72 | 0.780 |  | 0.41 | 0.508 |
| Ventilator-free days | 1.0 | 1.0 |  | 0.17 | 0.295 |

Abbreviations: ICU, intensive care unit; EN, Enteral nutrition; PN, Parenteral nutrition; IQR, interquartile range.
